# Effect of an HPV Vaccination Multi-Level, Multi-Component Program on HPV Vaccination Initiation and Completion in a Pediatric Clinic Network

**DOI:** 10.3390/vaccines12050510

**Published:** 2024-05-08

**Authors:** Lara S. Savas, Ross Shegog, Erica L. Frost, C. Mary Healy, Dale S. Mantey, Sharon P. Coan, L. Aubree Shay, Travis A. Teague, Juan J. Ferreris, Sharice M. Preston, Sally W. Vernon

**Affiliations:** 1 Center for Health Promotion and Prevention Research, UTHealth Houston School of Public Health, Houston, TX 77030, USA; ross.shegog@uth.tmc.edu (R.S.); erica.l.frost@uth.tmc.edu (E.L.F.); sharoncoan@comcast.net (S.P.C.); travis.a.teague@uth.tmc.edu (T.A.T.); sally.w.vernon@uth.tmc.edu (S.W.V.); 2 Department of Pediatrics, Infectious Diseases Section, Baylor College of Medicine, Houston, TX 77030, USA; chealy@bcm.edu; 3 Michael & Susan Dell Center for Healthy Living, UTHealth Houston School of Public Health in Austin, Austin, TX 78701, USA; dale.s.mantey@uth.tmc.edu; 4 Center for Health Promotion and Prevention Research, UTHealth Houston School of Public Health in San Antonio, San Antonio, TX 78229, USA; laura.aubree.shay@uth.tmc.edu; 5 Christus Health, Children’s General Pediatric Clinic, San Antonio, TX 78257, USA; juan.ferreris@christushealth.org

**Keywords:** human papillomavirus, HPV vaccination, clinic-based multi-level intervention, implementation, quality improvement, adolescents

## Abstract

Despite clear evidence of the public health benefits of the human papillomavirus (HPV) vaccine in preventing HPV-related cancers and genital warts, underutilization of HPV vaccination in the United States persists. Interventions targeting multi-level determinants of vaccination behavior are crucial for improving HPV vaccination rates. The study’s purpose was to implement and evaluate the adapted Adolescent Vaccination Program (AVP), a clinic-based, multi-level, multi-component intervention aimed at increasing HPV vaccine initiation and completion rates in a five-clinic pediatric network in Bexar County, Texas. The adaptation process was guided by established frameworks and involved formative work with clinic stakeholders. The study utilized a quasi-experimental single group pre- and post- study design, with an external comparison data using the National Immunization Survey-Teen (NIS-Teen) datasets for the same time period to examine the AVP’s effect on HPV vaccination initiation and completion. A series of interrupted time series analyses (ITSA) compared the clinic system patient outcomes (HPV vaccination initiation and completion rates) in the post-intervention to the general adolescent population (NIS-Teen). Of the 6438 patients (11–17 years) with clinic visits during the 3-year study period, HPV vaccination initiation rates increased from 64.7% to 80.2% (*p* < 0.05) and completion rates increased from 43.2% to 60.2% (*p* < 0.05). The AVP was effective across various demographic and economic subgroups, demonstrating its generalizability. ITSA findings indicated the AVP improved HPV vaccination initiation and completion rates in clinic settings and that AVP strategies facilitated resilience during the pandemic. The minimal adaptation required for implementation in a new clinic system underscores its feasibility and potential for widespread adoption.

## 1. Introduction

The public health benefit of the human papillomavirus (HPV) 9-valent vaccine is clear in its opportunity to prevent infection of 9 HPV types, which cause an estimated 90% of cervical and anal cancers [1,2,3], 70% of oropharyngeal cancer [4], 75% vaginal cancer [2], 69% of vulvar cancers [2], 63% of penile cancers [2], and 90% of genital warts [2,5]. By preventing nine high-risk HPV infections, the HPV 9-valent vaccine has the potential to prevent approximately 34,400 new cases of HPV-associated cancers in the United States (U.S.) each year [6]. However, each year in the U.S., approximately 196,000 women are diagnosed with high-grade cervical dysplasia [7], 360,000 men and women are affected by genital warts [8], 1.4 million women are diagnosed with low-grade cervical dysplasia [9], and over three million abnormal cervical cancer Pap screenings [9,10]. While the Centers for Disease Control and Prevention (CDC) [11], American Cancer Society [12], and American Academy of Pediatrics [13] recommend completion of the HPV vaccination series by 13 years of age to prevent HPV-related cancers and related morbidity in males and females, HPV vaccine initiation rates fall far below other adolescent vaccines recommended at the same age (i.e., Tdap and meningococcal) [14]. Moreover, after 2018, HPV vaccine series completion, or HPV-up-to-date (HPV-UTD: >3 doses or >2 doses when the first dose was initiated before age 15 years) rates, have stagnated [14] with most states attaining below 50% HPV-UTD rates [15]. In 2022, the national HPV-UTD rate for males 13–17 years (60.6%) [16] and females 13–17 years (64.6%) [16] was far below the Healthy People 2030 goal of 80% [17].

HPV vaccination coverage (with >1 dose) and completion rates differ by ethnicity/race [18]. Minority Hispanic/Latino, Black, and American Indian/Alaskan Native youth have demonstrated greater coverage compared to non-Hispanic (NH) White and NH Asian youth [16,19]. While minority HPV-UTD rates have historically been reported as lower [20], most recent data suggest the NH White HPV-UTD rates now lag behind all minority groups, except for female Hispanics [21]. 

System-, provider-, and parent-level barriers to HPV vaccination include lack of consistent patient reminders of when vaccination is due, lack of clinician awareness of vaccine-eligible youth and associated missed opportunities, lower parental knowledge about vaccine dosing or scheduling, and inadequate patient-provider communication that alleviates vaccine hesitancy [14]. Evidence for system-, provider- and parent-targeted strategies to mitigate these barriers has been established. Strategies include system-level changes to electronic health records to routinize provider prompts to recommend HPV vaccination and audit and feedback strategies [22], provider-level interventions to promote effective provider communication (including presumptive, bundled, and/or unqualified provider recommendations) [23], and patient reminders [24] and parent education [25] aimed at parent determinants. There is also modest evidence for the implementation of parent- and patient-focused apps to promote HPV education and vaccination [26,27,28]. 

**Figure 1:**
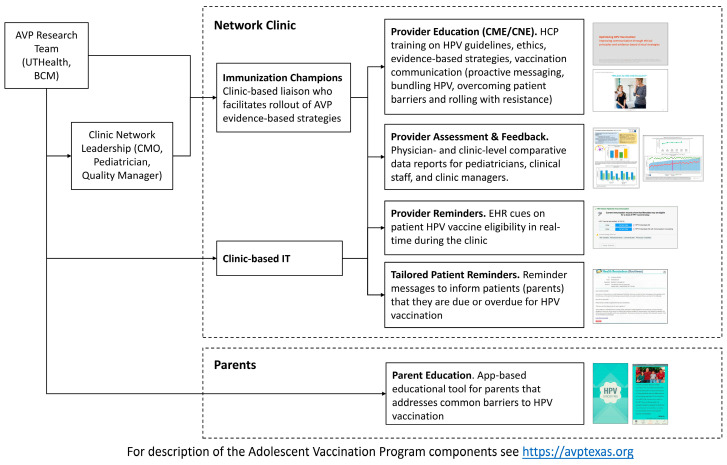
The Adolescent Vaccination Project (AVP) Clinic-Based Multi-level, Multi-Component Evidence-Based Strategies. Note Abbreviations: Information Technology (IT); Health Care Provider (HCP).

Emerging evidence suggests the strength of using multiple intervention strategies through multi-level and multi-component (MLMC) clinic-based intervention approaches to address the complex factors affecting vaccination at the clinic system (organization), provider, and parent levels [29,30,31,32]. Recent studies indicate that implementing these evidence-based interventions concurrently has a synergistic impact on HPV vaccination rates [33,34,35]. The Community Preventive Services Task Force (CPSTF), also known as the ‘Community Guide’, offers compelling evidence for the effectiveness of these intervention strategies in increasing vaccination rates [36]. Our team developed and evaluated the clinic-based MLMC intervention called the Adolescent Vaccination Program (AVP) to address the need for evidence-based MLMC HPV vaccination interventions designed for clinic organizations to implement to reach diverse patient populations. The AVP comprises six evidence-based strategies that target clinic systems, providers, and parents (Figure 1). AVP development was guided by the Intervention Mapping systematic approach [37], informed by behavioral theory (i.e., Social Cognitive Theory [38], Theory of Reasoned Action [39], Health Belief Model) [40], and guided by formative work with clinic leadership, providers (pediatricians and medical assistants) and parents [41], to increase provider delivery of consistent, bundled (when possible), presumptive and unqualified HPV vaccination recommendations [42].

We previously demonstrated that the AVP effectively increased HPV vaccination initiation in a 51-clinic pediatric network in Houston, Texas [43]. The patient population in the original study was primarily privately insured (80.3%), identified as English speakers (93.1%), and was demographically diverse (44.4% non-Hispanic White, 24.8% Hispanic and 13.8% Hispanic, and among 17% ethnicity was unknown). The purpose of this study was to adapt, implement, and evaluate the AVP’s effect on HPV vaccination initiation and completion in a five-clinic pediatric network. The new clinic setting comprised a primarily insured, English-speaking, and largely diverse patient population, with a slightly larger proportion of Hispanic/Latino patients, reflecting the local population in Bexar County, Texas. This study was designed to increase understanding regarding the generalizability of the AVP and to contribute new evidence regarding its effect on increasing the completion of the HPV vaccination series.

## 2. Materials and Methods

### 2.1. The Evidence-Based AVP HPV Vaccination Intervention

The AVP is a pediatric clinic-based intervention that comprises six evidence-based strategies aimed at the clinic organization, providers and parents and designed to synergistically facilitate an increase in HPV vaccination initiation and completion, including (1) HPV immunization champions [44], (2) provider assessment and feedback (A&F) [22,45], (3) continuing medical/nursing education (CME/CNE) [46], (4) electronic health record (EHR)-based provider reminders [47], (5) patient (parent) HPV vaccination reminders [48], and (6) a self-tailored parent education app [49,50]. 

The champions facilitate the implementation of AVP strategies in their clinic (e.g., by distributing A&F reports, promoting continuing education activity to all clinic staff, training clinic staff on provider and parent reminders, and distributing HPV vaccination education materials for parents in clinic examination and waiting rooms). The provider A&F quarterly reports provide a comparison of each provider’s and clinic’s HPV vaccination initiation and completion statistics for the previous quarter and HPV vaccination goals for the coming quarter to raise provider awareness of personal and clinic HPV vaccination rates. The online 50 min ethics-accredited CME/CNE HPV vaccination communication education included provider and clinical staff training on HPV vaccine national guidelines, evidence-based strategies to increase HPV vaccination, and communication strategies for providers to deliver strong, presumptive [51], bundled (when concurrent with other vaccines) HPV vaccination recommendations, and to roll with patient resistance [52]. The CME/CNE engaged viewers with real-life case vignettes aimed at enhancing provider communication skills and confidence to deliver strong recommendations. Provider EHR-based real-time HPV vaccination reminders are designed to be delivered during the patient encounter to reduce missed vaccination opportunities. The AVP team worked with the clinic system staff to design reminder messages and coordinate delivery through the EHR system (i.e., Epic software). 

The clinic-wide patient registry and HPV vaccination reminder system were developed to notify parents of their child’s vaccine initiation due date (triggered by birth date) and to remind parents of completion due dates for the 2nd (or 3rd) HPV vaccination dose. This system was designed for automated delivery through the Epic MyChart patient portal. The parent educational materials promoted positive messaging about the HPV vaccine, as well as provided a QR code for parents to download the AVP *HPVcancerFree* parent education app. Outcome analyses of the original AVP conducted in a 51-pediatric clinic network indicated the AVP MLMC intervention approach effectively increased HPV vaccination initiation 28% from baseline to 3-year follow-up compared with a 20% change for Houston reported by the NIS-Teen data for the same age group and period (2014 through 2018) [43].

### 2.2. The Adapted AVP Intervention

In the current study, our team, comprising UTHealth behavioral scientists and a Baylor College of Medicine pediatric infectious disease specialist, adapted, implemented, and evaluated the AVP intervention for a new five-clinic pediatric network. Adaptation was guided by the Intervention Mapping approach and CDC Replicating Effective Programs (REP) frameworks [37,53]. The REP framework includes four phases: (1) preconditions (to identify need, target population, and suitability of the intervention); (2) pre-implementation activities (e.g., adapting the program for the new practice setting and patient population with stakeholder input and planning implementation rollout with clinic leadership); (3) implementation (e.g., delivery of the AVP strategies); and (4) maintenance (e.g., preparing the AVP intervention for sustainability). 

Focus groups and surveys were conducted with clinic staff and managers to identify psychosocial factors in patients and providers (e.g., social and cultural norms) and clinic structural factors (e.g., EHR capabilities) that affect the implementation of the clinic-based AVP strategies. We did not identify new cultural-related determinants of vaccination that required adaption to parent education. Adaptation included updates to CME and CNE content, including updated HPV vaccination guidelines, new information on HPV vaccine safety, and more evidence for vaccinating at the recommended age (11–12 years). Working with the clinic partners, we identified the need to tailor the A&F preparation strategy to fit with the clinic Athena EHR system capabilities. In addition, the delivery of A&F reports to each provider was designed to align with the clinic organization. Reports were compiled by the AVP team and delivered to champions via email. Champions at each clinic site printed the reports and distributed them to physicians and other clinic staff. 

To adapt and integrate the provider reminder strategy into the new clinic system EHR platform, the AVP team worked with the medical director and EHR team. The result was the creation of provider HPV vaccination job aids that outline HPV vaccination best practices and instructions for providers that included the following steps to set up vaccination reminder prompts: (1) use the EHR’s (i.e., Athena) quality tab to check whether the patient is due for HPV, (2) subscribe to a customized order set that includes HPV, and (3) manually add HPV immunization alerts to patient’s records. These job aids were distributed to all physicians and managers. Champions and managers promoted the job aids and posted them in exam rooms for easy reference by physicians and clinical staff. 

Parent reminder delivery methods were also adapted to align with the clinic patient communication infrastructure, changing the delivery method, which was developed with the previous clinic system [43], to sending reminders through an automated email reminder campaign to align with the clinic system’s current patient communication methods. The AVP *HPVcancerFree* parent education app adaptation was informed by the formative work with the new system adoptees. In addition, we reviewed the parent survey and app usage data from the original AVP evaluation to improve user engagement and improved relevance to parents. AVP *HPVcancerFree* parent education app adaptations included the addition of (1) an animated, infographic-based video on HPV and the HPV vaccine, and (2) biweekly push notifications containing factual information to serve as reminders to app users and as an additional source of information. The AVP intervention strategies are presented in Figure 1 below and may be viewed at the AVP URL https://avptexas.org (accessed on 29 April 2004).

### 2.3. Study Setting and Population

Eligible clinics were part of an urban pediatric clinic network in Bexar County, Texas, which serves approximately 7000 children and adolescents aged 11–17 years per year, and served approximately 26,000 11–17-year-olds from 2018 to 2022. No clinics in this system were excluded. At the time of AVP intervention implementation, the system had five clinic sites, 18 pediatricians, and 40 clinical staff (e.g., CMA, RN, LVN). Of those patients, most were privately insured (85%) and identified as Hispanic. (Table 1). 

### 2.4. Study Design

We conducted a quasi-experimental single group, pre- and post-study design with an external comparison group to examine the effect of the adapted AVP intervention on HPV vaccination initiation and completion rates and compare the HPV vaccination initiation rates to the original AVP study. This research was approved by the Institutional Review Board at the University of Texas Health Sciences Center at Houston (HSC-SPH-18-0733). 

### 2.5. Measures

The primary outcomes include HPV vaccination initiation among 11–12-year-olds and HPV vaccination completion, or up-to-date (UTD), by age 13. UTD status is defined as having ≥3 doses, or 2 doses when the first HPV vaccine dose was initiated before age 15 years of age and there were at least 5 months between the first and second dose. We also examined HPV vaccination among 13–17-year-olds to examine the effect of the intervention on the catch-up age groups. We ascertained HPV vaccination outcomes from EHR data at baseline and follow-up. Patient sociodemographic characteristics were also obtained through EHR data, including age (11–12 and 13–17), sex, race/ethnicity (NH Black, Hispanic/Latino, NH White), and health insurance status (public or private). 

### 2.6. Comparison Data

Comparison data are from the National Immunization Survey-Teen (NIS-Teen) datasets for the years 2017–2021, which provides vaccination surveillance data based on a representative sample of adolescents 13–17 years of age, collected among U.S. parents (mailed questionnaires) and providers (by telephone) [54]. The NIS-Teen dataset includes HPV vaccination uptake (≥1 dose) and vaccination UTD rates (≥3 doses, or 2 doses when the first HPV vaccine dose was initiated before age 15 years of age and there were at least 5 or more months minus 4 days, between the first and second dose). The age group reported by NIS-Teen was 13–17 years so we used that age group as our comparison to examine secular trends. The NIS-Teen database presents coverage rates (≥1 dose of HPV vaccine) based on a representative sample of males and females nationally (sample sizes in the years 2017–2021 range from 990–2256 in Texas and 296–336 in Bexar County, Texas. 

### 2.7. Statistical Methods

Before testing the study hypotheses, we calculated patient-level mean and prevalence figures (i.e., descriptive statistics) for study variables among all patients presenting for care during the study period. To test the study hypotheses, we conducted a series of interrupted time series analyses (ITSA) [55,56]. First, we used an ITSA to compare trends in HPV vaccine initiation before delivery of the intervention (1 September 2017 to August 2018) and relative to after delivery of the intervention (1 September 2018 to 31 May 2022). Second, we conducted a similar ITSA to compare trends in HPV vaccine competition before delivery of the intervention, relative to after delivery of the intervention. This method is used to compare the outcome (HPV vaccination initiation) in the post-intervention among the clinic system patient population period to the counterfactual [57]. Each ITSA controlled for age, sex, race/ethnicity, and health insurance status. We also conducted an interaction between interruption and race/ethnicity to test for any possible differences in treatment effect. In addition, we conducted a comparison of clinic HPV initiation and completion (UTD) trends with trends in the general adolescent population provided by NIS-Teen data (2017–2021). All analyses were conducted in Stata 17.2 (College Station, TX, USA).

## 3. Results

During this three-year intervention study, an average of 6771 patients, aged 11 through 17 years, completed a clinical appointment (52.1% female and 47.9% male). Patients’ sociodemographic characteristics, as ascertained from EHRs, indicate that 43% of patients were identified as Hispanic, 33.8% NH White, and 4.3% NH Black. Patients were primarily covered by private insurance, with 10.4% covered by Medicaid, and the majority spoke English (Table 1).

### AVP Intervention Effect on HPV Initiation and Completion Rates

**Figure 2:**
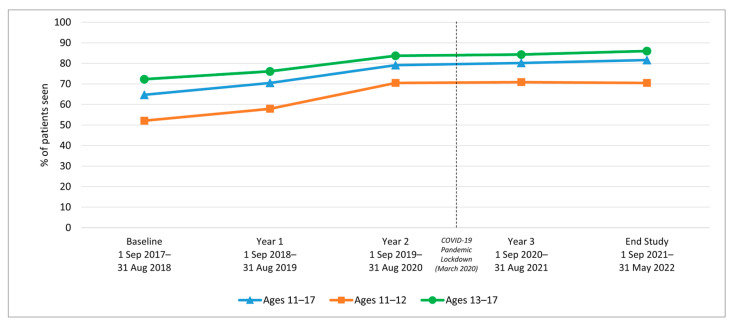
HPV Vaccine Initiation for Clinic System Patients by Age Group, 2017–2022.

**Figure 3:**
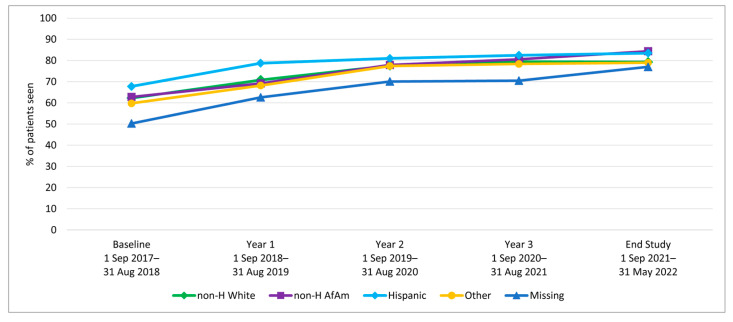
HPV Vaccine Initiation by Race/Ethnicity for Clinic System Patients Aged 11–17.

**Figure 4:**
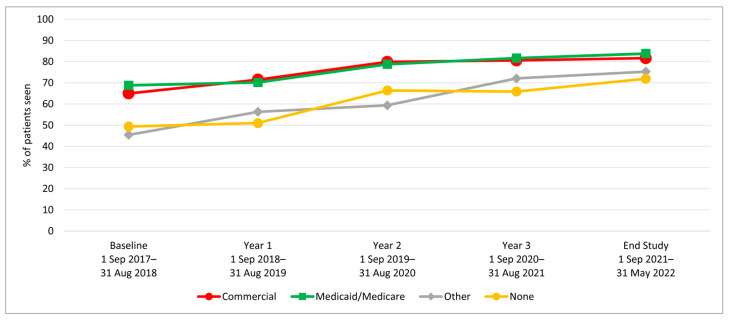
HPV Vaccine Initiation by Patients’ Insurance Type Aged 11–17.

We examined HPV vaccination initiation and completion trends from the baseline period (1 September 2017–31 August 2018) to year 1, before rolling out the AVP intervention strategies, and annually through year 3 (1 September 2020–31 August 2021), as well as at a 9-month post-intervention follow-up period. Results indicate a significant increase in initiation rates among 11–17-year-olds from baseline to 3-year follow-up (64.7% to 80.2%; *p*-value < 0.001) (Table 1). Similarly, initiation rates increased from baseline to the 3-year follow-up among both younger adolescents (11–12-year-olds: 52.1% to 70.9%) and older adolescents (13–17-year-olds: 72.3% to 84.3%) (Figure 2). Initiation rates also increased for patients across all races/ethnicities and insurance coverage types, including those with commercial insurance, Medicare/Medicaid, and those with no insurance (Table 1 and Figure 3 and Figure 4). 

**Figure 5:**
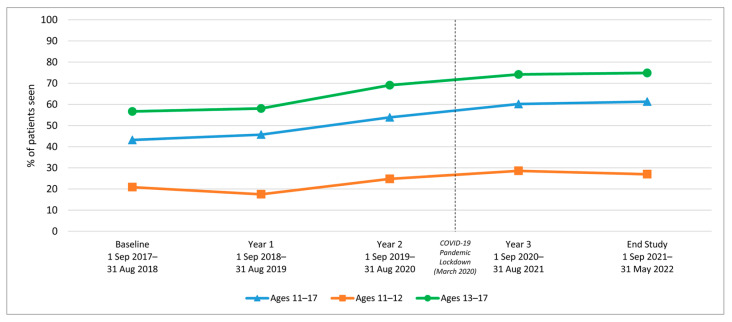
HPV Vaccine Completion for Clinic System Patients by Age Group, 2017–2022.

**Figure 6:**
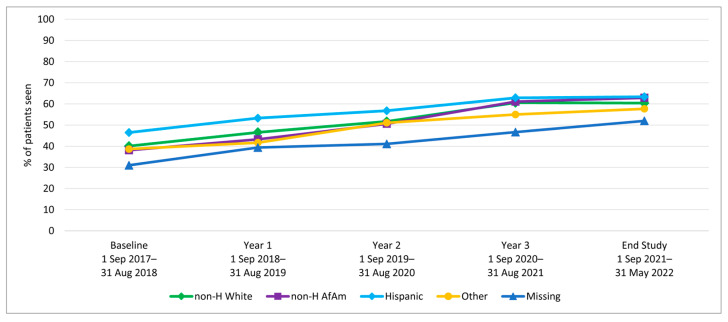
HPV Vaccine Completion by Race/Ethnicity for Clinic System Patients Aged 11–17.

**Figure 7:**
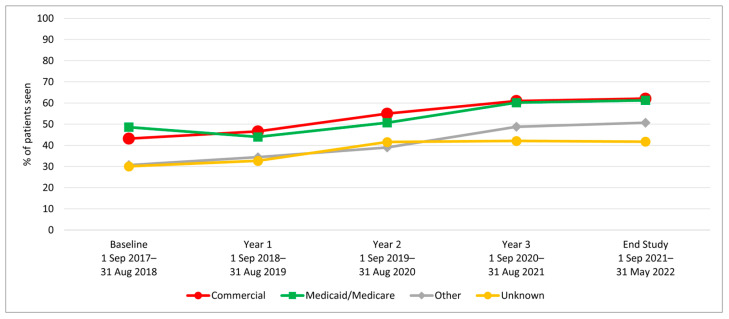
HPV Vaccine Completion by Patients’ Insurance Type Aged 11–17.

HPV vaccination completion rates also increased significantly among 11–17-year-olds from baseline to the 3-year follow-up (43.2% to 60.2%; *p*-value < 0.001) (Table 2). The increase was observed among both younger adolescents (11–12-year-olds: 20.9% to 28.6%) and older adolescents (13–17-year-olds: 56.7% to 74.2%) (Figure 5). In addition to age, the increase over time was observed across all race/ethnicity and insurance groups (Table 2 and Figure 6 and Figure 7).

**Figure 8:**
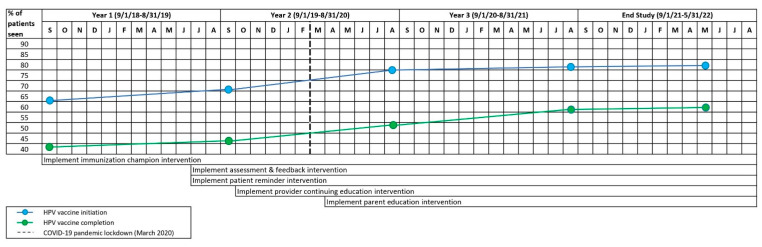
Increase HPV Initiation and Completion Among Patients Aged 11–17 Years, by Year and Adolescent Vaccination Project (AVP) Intervention Strategy Roll-Out.

Overall, a greater increase in initiation and completion rates occurred from year 1 to year 2, following the rollout of A&F, patient reminders, provider CME/CNE, and the parent education app (Figure 8).

**Figure 9:**
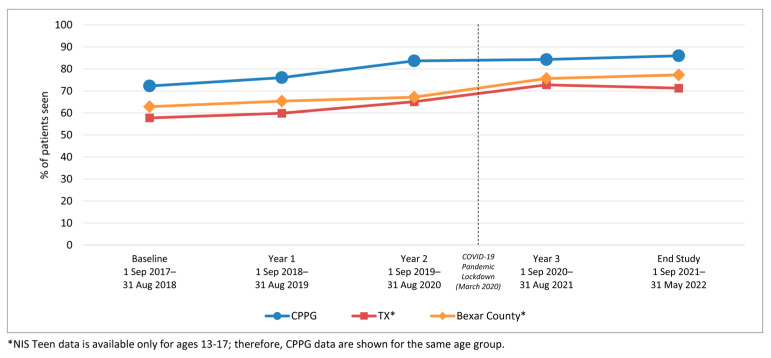
HPV Vaccine Initiation Comparing Intervention Clinics, Texas, and Bexar County Rates, Ages 13–17 *.

**Figure 10:**
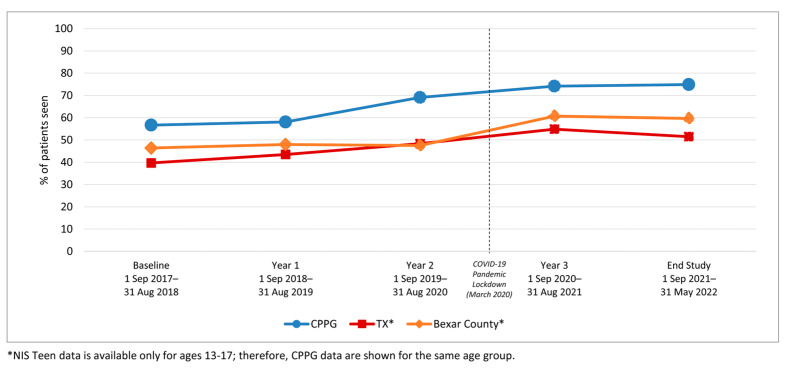
HPV Vaccine Series Completion Comparing Intervention Clinics, Texas, and Bexar County Rates, Ages 13–17 *.

Figure 9 and Figure 10 present HPV vaccination rates comparing the intervention clinic network patient population initiation and series completion rates to adolescents in the same age groups residing in Bexar County (where the clinic system is located) and Texas. The external comparison was based on NIS-Teen data provided for 13–17-year-olds in the same period (January 2017–February 2022) [58,59,60].

**Figure 11:**
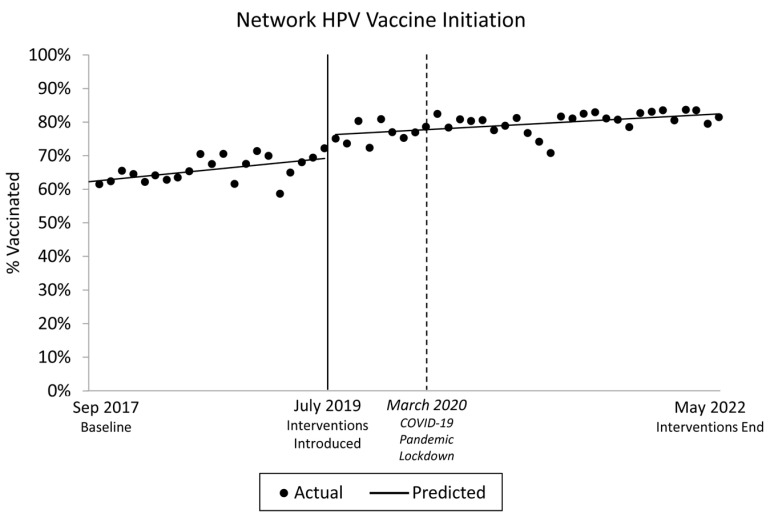
Interrupted Time Series for the Five-Clinic Network, 2017–2022.

Figure 11 presents the ITSA of the network HPV vaccination initiation data results, which indicate that prior to the intervention, HPV vaccine initiation prevalence was significantly increasing at ~0.34% per month (*p* = 0.002), relative to ~0.18% after the intervention. These trends were not statistically different (*p* = 0.173); however, the onset of the intervention (i.e., the “interruption”) was associated with a significant increase in HPV vaccine initiation (+3.0%; *p* = 0.002). No statistical differences were observed between race/ethnicity categories with Hispanic as the referent group or NH White as the referent group. 

**Figure 12:**
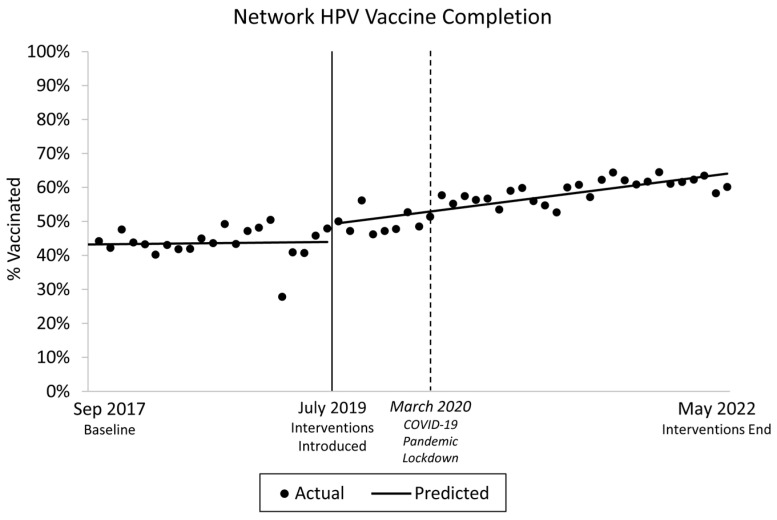
Interrupted Time Series for the Five-Clinic Network, 2017–2022.

Figure 12 presents HPV vaccination completion ITSA results, which indicate that prior to the intervention, HPV vaccination completion was unchanged (~0.04% per month; *p* = 0.812) and then increased to 0.43% per month following the intervention (*p* < 0.001). These trends were significantly different, indicating that HPV vaccine competition increased significantly following the intervention (*p* = 0.033). There was no immediate change in the prevalence of HPV vaccination completion (*p* = 0.077) at the onset of the intervention (i.e., interruption).

Because the COVID-19 pandemic lockdown was initiated on 15 March 2020 [61], we assessed the potential effect of the pandemic on HPV vaccination rates during the 3-year intervention period. We found no decrease in HPV vaccination when comparing HPV vaccination rates pre-COVID with post-COVID within the five-clinic network. 

## 4. Discussion

To improve vaccination efforts, we developed the AVP, comprised of evidence-based strategies [48,62,63,64] to address clinic system-, provider- and patient-related factors influencing HPV vaccination in clinic settings (Figure 1). In our first evaluation of the MLMC intervention (2015–2017), in a 51-clinic network with 44.4% NH White, 24.8% Hispanics, and 13.8% NH Black patients, and predominately English speakers (93.1%), we found the AVP effectively increased HPV initiation rates among 11–17-year-olds by 36% from baseline to 3-year follow-up across all clinic sites [43]. In this current study (2018–2022), in the five-clinic network with fewer NH White (33.8%) and NH Black patients (4.3%), a larger proportion of Hispanic patients (43%), and a slightly higher proportion of English speakers (95.8%), we found the adapted AVP again effectively increased HPV vaccination initiation rates by 24% from baseline to 3-year follow-up, to an overall 80% initiation among 11–17-year-olds. However, we observed the rates began to plateau once clinic site initiation rates approached 80%, indicating there could be a threshold effect. Additionally, this current study establishes the effectiveness of the AVP on increasing HPV vaccination completion rates from 43.2% at baseline to 60.2% at 3-year follow-up (while varying, an increase was observed across each subgroup) (Table 2). Comparing clinic initiation and completion ITSA rates with those among adolescents in the same age groups residing in Bexar County during the same period, we observed that the onset of the intervention was associated with a significant increase in initiation prevalence (+3.0%; *p* = 0.002). In addition, we observed a 0.43% per month increase in completion rates following the intervention (*p* < 0.001). No differences in treatment effect were found by race/ethnic subgroups, indicating that the positive effect of the AVP intervention was observed across race/ethnicity groups. This replicates the findings of our original AVP effectiveness study focused on increasing HPV vaccination initiation [43] and adds new findings regarding effectively increasing completion rates. 

The success of the AVP also indicates that minimal adaptation of the MLMC AVP program for the new clinic system was needed to retain its effectiveness on initiation, as well as to positively affect HPV vaccination completion rates. This study thus provides strong evidence of feasibility regarding the minimal burden on AVP program clinic adopters to adapt the AVP strategies for new clinic settings and implement them with fidelity. Thus, the results from this study indicate promise regarding the generalizability of the strategies and scalability of the AVP approach. Moreover, this study adds to the evidence in the literature regarding using MLMC clinic-based intervention strategies [65], including training HPV vaccination champions to implement provider reminder job aids and assessment and feedback (A&F), encouraging providers to complete the CME/CNE to obtain HPV vaccination-specific communication training focused on increasing providers’ consistent and effective HPV vaccination recommendation style with adolescent patients/parents and delivering parent education via an HPV vaccination self-tailored app [66].

Importantly, during this study, the COVID-19 pandemic shutdown in the U.S. began in mid-March 2020. During the early pandemic period that coincided with this study, the vaccination initiation and completion rates in the AVP clinic sites continued to rise, albeit with slightly depressed increases (Figure 2, Figure 5, Figure 8 and Figure 9). This increase occurred despite a marked decline in pediatric vaccine administration during the COVID-19 pandemic [67,68]. Moreover, while the NIS-Teen data indicate a significant decrease in HPV vaccination initiation rates among Medicaid and uninsured adolescents during the pandemic period [16], in the present study, these groups continued to experience a slight increase in both initiation and completion rates, suggesting the AVP strategies mitigated the COVID-19-related decline in HPV vaccination rates. Findings of AVP effectiveness during the pandemic were supported by a smaller HPV vaccination quality improvement project conducted in two clinics during the pandemic, which also found the HPV vaccination strategies helped to sustain both HPV initiation and completion rates during the pandemic [69].

This study also has several limitations, particularly related to study design. We used a one-group pre- and post-study design, and the analysis methods did not control for time-varying confounders [57]. We also cannot control for secular trends; however, during the pandemic, secular vaccination trends would not have supported an increase in vaccination rates, and rather, our examination of the vulnerable subgroups most affected by the pandemic (uninsured and Medicaid-covered adolescents) suggests the AVP mitigated negative effects on these vulnerable patient groups receiving care at the AVP clinics. Finally, implementation of the AVP was partially supported by the UTHealth research team, particularly in facilitating the adaptation of the AVP strategies to fit with the capacity and EHR infrastructure of the adopting clinic system. This study also has several strengths. First, this study replicates the original AVP’s effect on increasing HPV vaccination initiation, as well as provides new evidence that the AVP had a significant effect on increasing HPV completion rates during the COVID-19 pandemic. By replicating the AVP in a smaller clinic network in a different city, with different patient population demographic profiles, EHR systems, and organizational factors, the results provide insight into the generalizability of the AVP approach across clinic systems. The HPV vaccination outcome data were also ascertained using EHR data. 

Findings from this intervention study further establish the AVP as an EBI that supports an increase in HPV vaccination initiation and completion in a pediatric clinic network. This study also serves as an example of the minimal adaptation required to prepare the AVP for new clinic systems. Finally, our findings suggest that the AVP vaccination strategies facilitated resilience during the pandemic, helping to support best practices to promote HPV vaccination initiation and completion. To promote scale-up and sustainment of the evidence-based AVP, research is underway to design implementation support strategies to guide clinic leaders and staff in safety-net clinic systems in the adoption, implementation planning, and delivery of the AVP [70].

## Figures and Tables

**Table 1:** Demographics and HPV Vaccine Initiation by Year, Patients Aged 11–17 Years.

Characteristics	Mean Number of Patients per Year	% of Patient Pop.	Baseline		Year 1		Year 2		Year 3		End of Study	
			N	%	N	%	N	%	N	%	N	%
Total	6771		6438	64.7	6703	70.5	6715	79.1	7229	80.2	5469	81.6
Ages 11–12	2253	33.3	2430	52.1	2048	57.9	2304	70.5	2228	70.9	1551	70.5
Ages 13–17	4519	66.7	4008	72.3	4655	76.0	4410	83.7	5001	84.3	3918	86.0
Female	3526	52.1	3387	66.6	3498	72.1	3482	79.7	3737	81.6	2756	83.5
Male	3245	47.9	3051	62.6	3205	68.7	3232	78.6	3492	78.6	2713	79.7
Non-Hispanic White	2290	33.8	1540	62.3	3692	70.8	1606	77.6	2322	79.4	1445	79.3
Non-Hispanic Black	292	4.3	231	62.8	319	69.3	267	77.9	350	80.6	256	84.4
Hispanic	2914	43.0	3739	67.8	823	78.7	3846	81.0	3249	82.5	2860	83.5
Other	860	12.7	602	59.8	1339	68.2	620	77.4	878	78.4	589	79.0
Missing or Unknown	415	6.1	326	50.3	530	62.6	375	70.1	430	70.5	319	77.4
Commercial Insurance	5774	85.3	5571	65.0	5723	71.5	5734	79.9	6069	80.6	4486	81.7
Medicare/Medicaid	707	10.4	613	68.8	682	70.2	708	78.8	824	81.7	733	83.8
Other Insurance	120	1.8	88	45.5	96	56.3	123	59.4	172	72.1	154	75.3
None	170	2.5	166	49.4	202	51.0	149	66.4	164	65.9	96	71.9
English Language	6488	95.8	6265	65.1	6514	71.0	6504	79.4	6669	80.7	5264	81.7
Spanish Language	141	2.1	116	59.5	137	59.1	150	76.0	159	78.6	157	81.5
Other Language	3	0.0	1	100.0	0	-	7	14.3	4	100.0	1	100.0
Unknown Language	140	2.1	56	32.1	52	44.2	53	60.4	397	72.0	47	74.5

Baseline = 1 September 2017–31 August 2018; Year 1 = 1 September 2018–31 August 2019; Year 2 = 1 September 2019–31 August 2020; Year 3 = 1 September 2020–31 August 2021; End = 1 September 2021–31 May 2022.

**Table 2:** Sample Demographics and HPV Vaccine Series Completion * by Year, Patients Aged 11–17 Years.

Characteristics	Mean Number of Patients per Year	% of Patient Pop.	Baseline		Year 1		Year 2		Year 3		End of Study	
			N	%	N	%	N	%	N	%	N	%
Total	6771		6438	43.2	6703	45.7	6715	53.9	7229	60.2	5469	61.3
Ages 11–12	2253	33.3	2430	20.9	2048	17.5	2304	24.8	2228	28.6	1551	27.0
Ages 13–17	4519	66.7	4008	56.7	4655	58.1	4410	69.1	5001	74.2	3918	74.9
Female	3526	52.1	3387	45.7	3498	47.7	3482	55.2	3737	61.6	2756	62.9
Male	3245	47.9	3051	40.4	3205	43.5	3232	52.5	3492	58.6	2713	59.7
Non-Hispanic White	2290	33.8	1540	40.1	3692	46.6	1606	51.7	2322	60.6	1445	60.4
Non-Hispanic Black	292	4.3	231	38.1	319	43.3	267	50.6	350	61.1	256	62.9
Hispanic	2914	43.0	3739	46.5	823	53.3	3846	56.8	3249	62.9	2860	63.4
Other	860	12.7	602	38.7	1339	41.7	620	51.1	878	55.0	589	57.7
Missing or Unknown	415	6.1	326	31.0	530	39.4	375	41.1	430	46.7	319	52.0
Commercial Insurance	5774	85.3	5571	43.2	5723	46.6	5734	55.0	6069	61.0	4486	62.1
Medicare/Medicaid	707	10.4	613	48.6	682	44.0	708	50.7	824	60.2	733	61.3
Other Insurance	120	1.8	88	30.7	96	34.4	123	39.0	172	48.8	154	50.7
None	170	2.5	166	30.1	202	32.7	149	41.6	164	42.1	96	41.7
English Language	6488	95.8	6265	43.4	6514	46.1	6504	54.5	6669	60.7	5264	61.5
Spanish Language	141	2.1	116	38.8	137	38.0	150	41.3	159	54.7	157	58.0
Other Language	3	0.0	1	100.0	0	-	7	0.0	4	25.0	1	100.0
Unknown Language	140	2.1	56	23.2	52	23.1	53	28.3	397	52.9	47	51.1

* Completion was defined as completing 2 doses by age 15 and 3 doses from 15–17 years of age. Baseline = 1 September 2017–31 August 2018; Year 1 = 1 September 2018–31 August 2019; Year 2 = 1 September 2019–31 August 2020; Year 3 = 1 September 2020–31 August 2021; End = 1 September 2021–31 May 2022.

## Data Availability

The data presented in this study are available on request from the corresponding author. The data are not publicly available due to security restrictions.

## Abbreviations

AVP	Adolescent Vaccination Program
A&F	Assessment and feedback
CDC	Centers for Disease Control and Prevention
CME/CNE	Continuing medical/nursing education
HER	Electronic health record
HCP	Health Care Provider
HPV	Human papillomavirus
IT	Information Technology
ITSA	Interrupted time series analyses
MLMC	Multi-level and multi-component
NIS-Teen	National Immunization Survey-Teen
REP	Replicating Effective Programs
U.S.	United States
UTD	Up-to-date
